# Impressic Acid Ameliorates Atopic Dermatitis-Like Skin Lesions by Inhibiting ERK1/2-Mediated Phosphorylation of NF-κB and STAT1

**DOI:** 10.3390/ijms22052334

**Published:** 2021-02-26

**Authors:** Jae Ho Choi, Gi Ho Lee, Sun Woo Jin, Ji Yeon Kim, Yong Pil Hwang, Eun Hee Han, Young Ho Kim, Hye Gwang Jeong

**Affiliations:** 1 College of Pharmacy, Chungnam National University, Daejeon 34134, Korea; chlkoala@naver.com (J.H.C.); ghk1900@cnu.ac.kr (G.H.L.); mpassword@cnu.ac.kr (S.W.J.); jykim525@o.cnu.ac.kr (J.Y.K.); yhk@cnu.ac.kr (Y.H.K.); 2 Subtropical/Tropical Organism Gene Bank, Jeju National University, Jeju 63243, Korea; 3 Fisheries Promotion Division, Mokpo City 58613, Korea; protoplast@hanmail.net; 4 Drug & Disease Target Research Team, Division of Bioconvergence Analysis, Korea Basic Science Institute (KBSI), Cheongju 28119, Korea; heh4285@kbsi.re.kr

**Keywords:** impressic acid, atopic dermatitis-like skin lesions, IgE, NF-κB, TARC

## Abstract

Impressic acid (IPA), a lupane-type triterpenoid from *Acanthopanax koreanum*, has many pharmacological activities, including the attenuation of vascular endothelium dysfunction, cartilage destruction, and inflammatory diseases, but its influence on atopic dermatitis (AD)-like skin lesions is unknown. Therefore, we investigated the suppressive effect of IPA on 2,4-dinitrochlorobenzene (DNCB)-induced AD-like skin symptoms in mice and the underlying mechanisms in cells. IPA attenuated the DNCB-induced increase in the serum concentrations of IgE and thymic stromal lymphopoietin (TSLP), and in the mRNA levels of thymus and activation regulated chemokine (TARC), macrophage derived chemokine (MDC), *interleukin-4* (IL-4), *interleukin-5* (IL-5), *interleukin-13* (IL-13), tumor necrosis factor-alpha (TNF-α) and *interferon-gamma* (IFN-γ) in mice. Histopathological analysis showed that IPA reduced the epidermal/dermal thickness and inflammatory and mast cell infiltration of ear tissue. In addition, IPA attenuated the phosphorylation of NF-κB and IκBα, and the degradation of IκBα in ear lesions. Furthermore, IPA treatment suppressed TNF-α/IFN-γ-induced TARC expression by inhibiting the NF-κB activation in cells. Phosphorylation of extracellular signal-regulated protein kinase (ERK1/2) and the signal transducer and activator of transcription 1 (STAT1), the upstream signaling proteins, was reduced by IPA treatment in HaCaT cells. In conclusion, IPA ameliorated AD-like skin symptoms by regulating cytokine and chemokine production and so has therapeutic potential for AD-like skin lesions.

## 1. Introduction

Atopic dermatitis (AD)-like skin diseases are the most common chronic inflammatory skin disorders due to innate and adaptive immune responses based on genetic, seasonal, and environmental causes [1]. AD-like skin diseases are characterized by pruritus, dry skin, abnormal immune responses, and IgE-mediated allergies that respond to various antigens [2]. The prevalence of these skin diseases is in millions of people around the world.

There are two hypotheses on the etiology of skin inflammation similar to AD-like skin disease. The first is primary immune dysfunction, leading to IgE sensitization and subsequent epithelial barrier disorders [3]. The second is an inherent genetic defect in the formation of the epidermal skin barrier, or blockage of the skin barrier due to environmental metastasis, causing skin symptoms similar to AD [4]. In addition, various internal and external factors contribute to the development of AD-like skin barrier dysfunction and immunomodulatory disorders [5].

Several commercial preparations for treating AD-like skin conditions are available as creams, gels, lotions, or ointments, but their effectiveness is limited [6]. In addition, there are several treatment regimens for AD such as topical glucocorticosteroids, calcineurin inhibitors, and immunosuppressive agents [7,8]. These remedies attenuate skin inflammation but have serious side effects. Topical steroids are the most effective treatment for AD-like skin lesions, but continued repeated application causes side effects [9]. Therefore, a new treatment without side effects is needed for AD-like skin symptoms. Various natural agents have been examined for the treatment of AD-like skin disorders.

Impressic acid (IPA; 3α-11α-dihydroxylup-20(29)-en-28-oic acid) is the first known lupane-type triterpenoid from *Schefflera impressa* [10]. In Korea, it is found in the leaves and roots of the *Acanthopanax koreanum* and is widely used as an herbal medicine for diseases such as hepatitis, rheumatism, type 2 diabetes, and inflammatory diseases [11,12]. IPA enhances endothelial nitric oxide synthase activation via the 5′ AMP-activated protein kinase, Ca^2+^ calmodulin-dependent protein kinase II, p38 MAPK, and the JNK1/2 pathways. In addition, IPA-induced nitric oxide production suppresses vascular inflammation by reducing intercellular adhesion molecule-1 expression and NF-κB activation [13]. IPA prevents cartilage degradation disorders by suppressing matrix metalloproteinase-13 expression [14]. IPA inhibits tumor necrosis factor-alpha (TNF-α) induced NF-κB activation and upregulates the transcriptional activity of peroxisome proliferator-activated receptor γ [15]. Recently, IPA reduced the LPS-induced inflammatory response in RAW264.7 macrophages [16]. Previous studies have shown the potential of IPA to attenuate inflammatory diseases including AD via suppressing NF-κB activation, but its effect and underlying mechanism on AD-like skin lesions have not been investigated. Therefore, we examined the inhibitory effect of IPA on 2,4-dinitrochlorobenzene (DNCB)-induced AD-like skin symptoms in BALB/C mice and the underlying mechanisms in HaCaT cells.

## 2. Results

### 2.1. IPA Attenuated DNCB-Induced AD-Like Skin Severity in Mice

**Figure 1:**
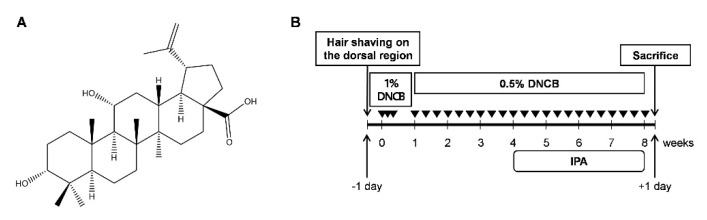
Schematic diagram of 2,4-dinitrochlorobenzene (DNCB)-induced atopic dermatitis (AD)-like skin lesions in BALB/C mice. (**A**) Chemical structure of impressic acid (IPA). (**B**) Schematic representation of the animal experiment.

The chemical structure of IPA is shown in Figure 1A. DNCB induces AD-like skin lesions in mice [17]. To evaluate whether IPA can attenuate AD-like skin lesions by DNCB in mice, BALB/C mice were topically treated with IPA after induction of AD-like skin lesions by DNCB (Figure 1B).

**Figure 2:**
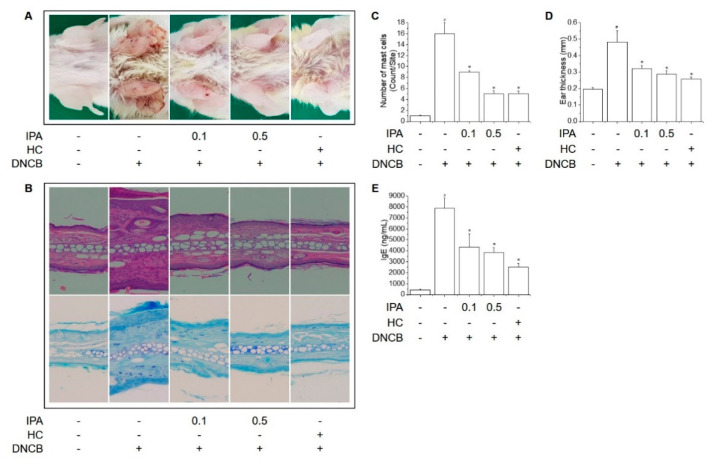
Inhibitory effect of IPA on DNCB-induced AD-like skin lesions in BALB/C mice. After inducing AD using DNCB, vehicle, IPA (0.1 and 0.5%, w/v) and HC (1%, w/v) was topically applied to the dorsal skin and ears of the mice six times per week for 4 weeks. (**A**) Representative image of AD-like ear lesions on the last day. (**B**) Histopathological analysis by H&E and toluidine blue staining of AD-like ear lesions. (**C**) Numbers of mast cells. (**D**) Ear thickness measured using a micrometer. (**E**) Serum IgE concentrations were assessed by ELISA. Results are means ± SD (n = 6). ^#^ *p* < 0.05, versus the control group; * *p* < 0.05, versus the DNCB-treated group. IPA: Impressic acid; HC: Hydrocortisone; DNCB: 2,4-dinitrochlorobenzene.

Repeated skin application of DNCB leads to AD-like skin severities, such as ear edema, scleroderma, and amassing of inflammatory and mast cells. In contrast, IPA alleviated these DNCB-induced symptoms (Figure 2A–D). Furthermore, IgE stimulates mast cells to release inflammatory cytokines in AD-like skin lesions [18,19]. Serum concentration of IgE was increased by DNCB but was significantly attenuated by IPA (Figure 2E).

### 2.2. IPA Attenuated DNCB-Induced Increased Levels of Thymic Stromal Lymphopoietin (TSLP) in Serum and Ear Lesions

**Figure 3:**
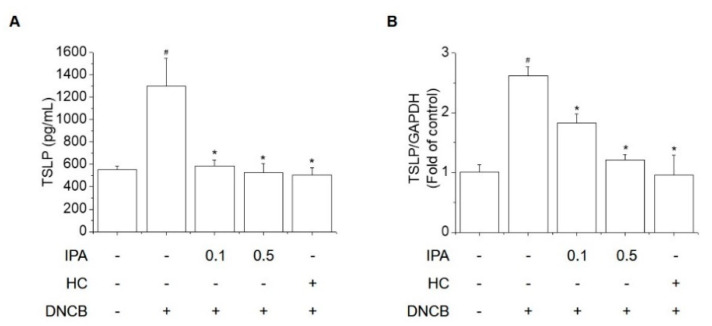
Inhibitory effect of IPA on the DNCB-induced increased serum concentration and mRNA level of thymic stromal lymphopoietin (TSLP) in BALB/C mice. (**A**) Serum TSLP concentrations were assessed by ELISA. (**B**) TSLP mRNA level was assessed by real-time PCR. Results are means ± SD (n = 6). ^#^ *p* < 0.05, versus the control group; * *p* < 0.05, versus the DNCB-treated group.

TSLP regulates both the onset and maintenance of AD-like skin symptoms and plays an important role in the activation and differentiation of Th2 cytokines, which trigger the secretion of IgE from AD-like skin lesions [20,21]. We examined the inhibitory effect of IPA on DNCB-induced TSLP levels in ear lesions and serum. The serum concentration and mRNA expression of TSLP were increased by DNCB but were significantly attenuated by IPA (Figure 3).

### 2.3. IPA Suppressed the DNCB-Increased mRNA Levels of Th1/Th2 Cytokines and Chemokines in Ear Lesions

**Figure 4:**
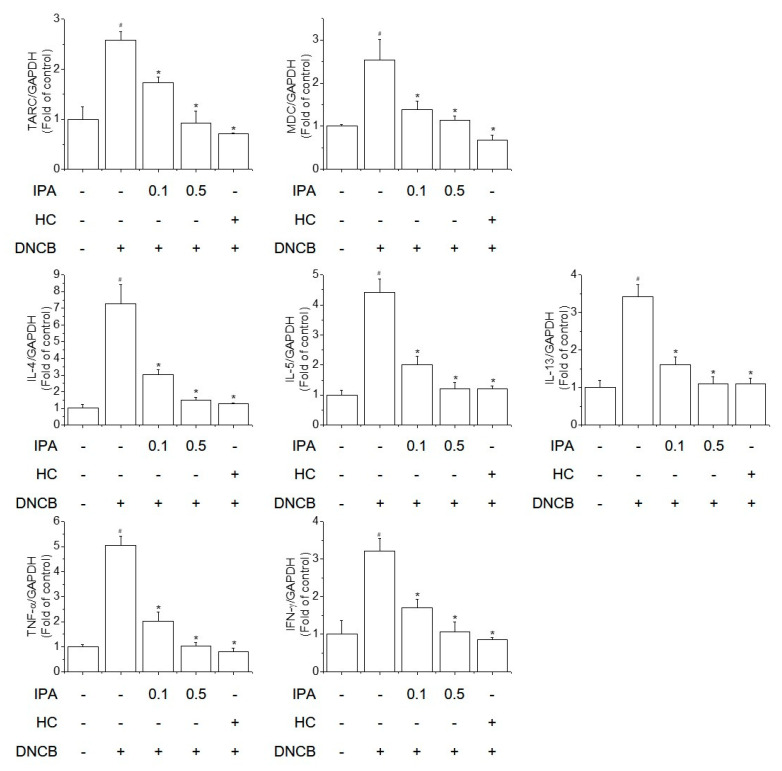
Inhibitory effect of IPA on the DNCB-induced increased mRNA levels of cytokines and chemokines in BALB/C mice. The mRNA levels of thymic stromal lymphopoietin (TARC), macrophage derived chemokine (MDC), interleukin-4 (IL-4), interleukin-5 (IL-5), interleukin-13 (IL-13), tumor necrosis factor-alpha (TNF-α), and IFN-γ were determined by real-time PCR. Results are means ± SD (n = 6). ^#^ *p* < 0.05, versus the control group; * *p* < 0.05, versus the DNCB-treated group.

According to a previous study, mast cells play a critical role in the secretion of pro-inflammatory mediators, including cytokines and chemokines [22]. IgE activates mast cells, enhancing pro-inflammatory cytokine expression in AD-like allergic skin lesions. AD-like skin symptoms are triggered by the interaction of Th1/Th2-predominant inflammation. Chemokines also play an important role in moving lymphocytes to the skin [23]. Thus, suppression of the production of these cytokines and chemokines may ameliorate AD-like skin diseases. We evaluated the effect of IPA on the mRNA levels of thymus and activation regulated chemokine (TARC), macrophage derived chemokine (MDC), interleukin-4 (IL-4), interleukin-5 (IL-5), interleukin-13 (IL-13), TNF-α, and interferon-gamma (IFN-γ) in ear lesions. IPA significantly reduced the mRNA levels of these Th1/Th2 cytokines and chemokines increased by DNCB (Figure 4). Therefore, IPA inhibits the expression of pro-inflammatory cytokines and chemokines, thus suppressing the symptoms of AD-like skin lesions, such as thickening of the dermis/epidermis and infiltration of inflammatory and mast cells.

### 2.4. IPA Inhibited the DNCB-Induced Increased Activation of NF-κB in Ear Lesions

**Figure 5:**
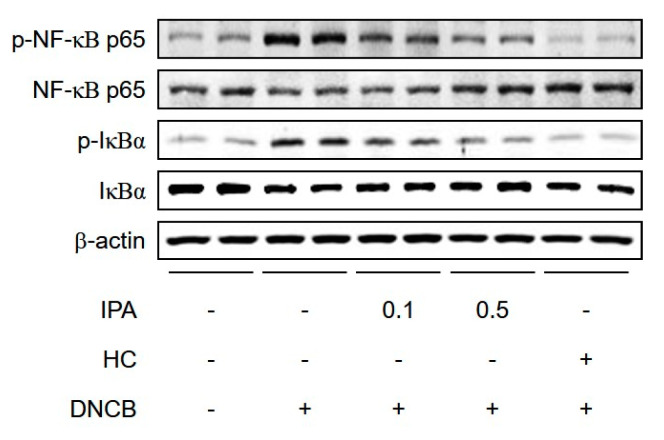
Inhibitory effect of IPA on DNCB-induced NF-κB activation in BALB/C mice. Protein levels of p-NF-κB p65, p-IκBα, IκBα, and β-actin were determined by Western blotting.

Cytokine secretion activated by T-cells is regulated by transcription factors, including NF-κB, which activate the expression of genes encoding pro-inflammatory cytokines and chemokines [24]. NF-κB is critical to the innate/adaptive immune response and inflammatory response, especially the Th1 response, and regulates inflammation caused by Th2 cell differentiation and activation [25]. We determined NF-κB activation in DNCB-induced mice by Western blotting. IPA significantly suppressed the phosphorylation of NF-κB and IκBα, and the degradation of IκBα, in DNCB-induced ear lesions (Figure 5). Therefore, IPA strongly inhibits the activity of NF-κB, a transcriptional regulator of pro-inflammatory cytokines and chemokines.

### 2.5. IPA Suppressed the TNF-α/IFN-γ-Induced Increased mRNA Levels of TSLP, TARC and MDC in Keratinocytes

**Figure 6:**
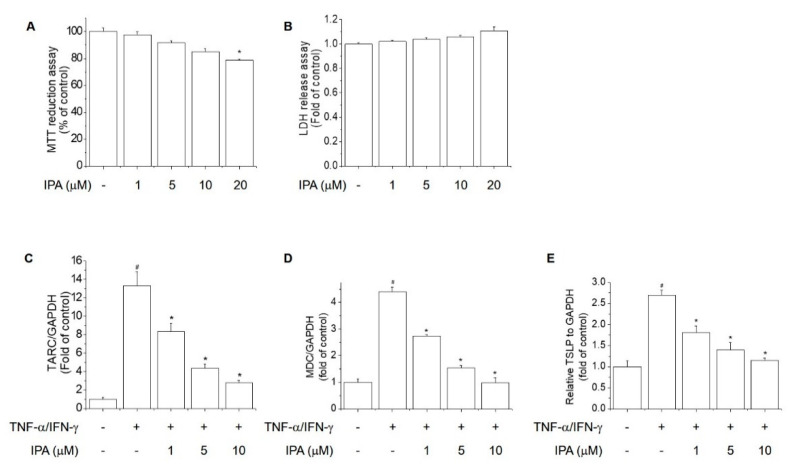
Inhibitory effect of IPA on the mRNA levels of TSLP, TARC, and MDC in TNF-α/IFN-γ-stimulated HaCaT cells. (**A**) Cells were treated with various concentrations of IPA for 24 h. Cell viability was assessed by MTT reduction assay. (**B**) Cell cytotoxicity was determined by LDH release assay. (**C**–**E**) Cells were treated with IPA (1, 5 or 10 μM) for 1 h, and then stimulated with TNF-α/IFN-γ (each 10 ng/mL) for 6 h, and the mRNA levels of TSLP, TARC, and MDC were determined by real-time PCR. Results are means ± SD (n = 3). ^#^ *p* < 0.01, versus the control group. * *p* < 0.01, versus the TNF-α/IFN-γ-treated group.

To evaluate the effect of IPA on cell viability and cytotoxicity, we performed the MTT reduction and LDH release assays in HaCaT cells. Cells were treated with various concentrations of IPA for 24 h. IPA at <10 μM had no significant effect on cell viability and cytotoxicity (Figure 6A,B). Keratinocytes play an important role in AD-like skin inflammation [26]. TSLP, TARC, and MDC are important in transporting lymphocytes to skin lesions [1,3,27]. Therefore, reducing TSLP, TARC, and MDC expression in keratinocytes may have therapeutic potential for AD-like skin lesions. Next, we performed the suppressive effect of IPA on the mRNA expression of these chemokines induced by TNF-α/IFN-γ in cells. The TNF-α/IFN-γ-induced increased mRNA levels of TSLP, TARC, and MDC were suppressed by IPA in HaCaT cells (Figure 6C–E).

### 2.6. IPA Inhibited TNF-α/IFN-γ-Induced NF-κB, MAPK, and STAT1 Activation in Keratinocytes

**Figure 7:**
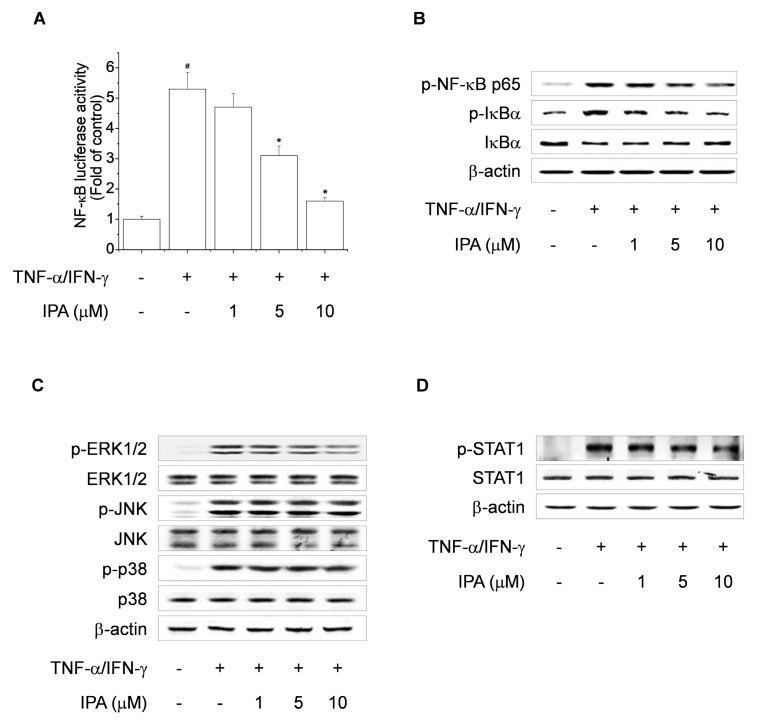
Inhibitory effects of IPA on activation of the NF-κB, extracellular signal-regulated protein kinase (ERK1/2), and STAT1 signaling pathways in TNF-α/IFN-γ-stimulated HaCaT cells. (**A**) Cells were transiently transfected with reporter plasmids containing tandem elements of NF-κB binding sites. After 5 h, the transfected cells were pretreated with IPA (1, 5, 10 μM), and then treated with TNF-α/IFN-γ (each 10 ng/mL) for 24 h, and luciferase activity was evaluated. Results are means ± SD (n = 3). Cells were treated with TNF-α/IFN-γ for 30 min in the presence of IPA. Protein levels of (**B**) p-NF-κB p65, p-IκBα, IκBα, and β-actin, (**C**) p-/t-MAPK and β-actin, and (**D**) p-/t-STAT1 and β-actin were assessed by Western blotting. ^#^ *p* < 0.01, versus the control group. * *p* < 0.01, versus the TNF-α/IFN-γ-treated group.

TNF-α/IFN-γ induces the release of chemokines and cytokines by activating NF-κB in skin keratinocytes. In addition, the NF-κB signaling pathway regulates the expression of TNF-α/IFN-γ-induced TARC in HaCaT cells [17]. To examine whether the influence of IPA was able to downregulate the on TNF-α/IFN-γ-induced TARC expression via the inhibition of NF-κB activation, we evaluated the effect of IPA on NF-κB promoter activity. Cells were transiently transfected with an NF-κB reporter vector. Pretreatment with IPA inhibited TNF-α/IFN-γ-induced NF-κB luciferase activity in a concentration–dependent manner (Figure 7A). To further evaluate the inhibitory effect of IPA on NF-κB activation, TNF-α/IFN-γ-stimulated HaCaT cell lysates were analyzed by Western blotting. IPA treatment suppressed TNF-α/IFN-γ-induced NF-κB p65 and IκBα phosphorylation and IκBα degradation (Figure 7B). MAPK is an important signaling factor in keratinocyte activation [28]. We next evaluated the inhibitory effect of IPA on TNF-α/IFN-γ-induced MAPK activation. TNF-α/IFN-γ induced the phosphorylation of extracellular signal-regulated protein kinase (ERK1/2), JNK1/2, and p38 MAPK in cells. However, IPA treatment selectively inhibited the activation of ERK1/2 (Figure 7C). STAT-1 has been implicated in AD-like skin-lesion-related signaling in TNF-α/IFN-γ-activated cells [29]. We evaluated the inhibitory effect of IPA on TNF-α/IFN-γ-induced STAT1 activation by Western blotting. TNF-α/IFN-γ activated phosphorylation of STAT1, but the effect was reduced by IPA treatment (Figure 7D). Therefore, IPA inhibits TNF-α/IFN-γ-induced TARC expression by selectively suppressing NF-κB, ERK1/2and STAT1 activation in HaCaT cells.

## 3. Discussion

AD-like skin disease is a multifactorial skin disease and has a complex relationship with innate and adaptive immune responses as well as with environmental, genetic, and psychological factors [30]. AD-like skin lesions show chronic, pruritic, and recurrent progression and affect 20% of children and 3% of adults worldwide [31]. AD-like skin disease, one of the most common chronic inflammatory skin diseases, is an IgE-mediated allergic disease of the epithelial barrier manifested by traumatic lesions, itching, dry skin, abnormal immune responses and various exogenous antigens [1].

AD-like skin diseases are typically treated with topical glucocorticosteroids, and cyclosporin A as gels, creams, or ointments, but these have limited effectiveness [6]. Such medications reduce skin inflammation but have serious side effects. However, there is increasing interest in natural product derived anti-inflammatory drugs for skin diseases because of the perception that such agents are inherently safe [17]. Recently, effort has focused on developing pharmaceuticals using natural ingredients for AD-like skin symptoms because of their low toxicity and high efficacy [32].

*A. koreanum* is an herb native to Korea, and its root and stem bark have been used to treat rheumatism and diabetes [33]. IPA is a triterpenoid component of the root of *Acanthopanax koreanum* and has marked anti-inflammatory activity [13,14,15,16,34]. However, its effect on AD-like skin lesions is unknown. Therefore, we assessed the inhibitory effect of IPA on AD-like skin lesions in BALB/C mice and in HaCaT cells.

The mechanisms underlying AD-like skin diseases are unknown but involve specific inflammatory and immune system responses mediated by IgE via a series of T helper cell interactions. The etiology of AD-like skin symptoms is allergic sensitization, exposure to allergens, overproduction of IgE and mast cells, Th1/Th2 imbalance, inflammation and infiltration of mast cells [35]. In this study, repeated topical application of DNCB exacerbated the symptoms of AD, such as ear lesions, ear swelling, inflammation, and infiltration of mast cells in mice, but these effects were ameliorated by IPA treatment.

Th1/Th2 imbalance and a defective skin barrier contribute to the progression of AD-like skin lesions [36]. Development of acute AD-like skin lesions involves a Th2-dominant inflammatory response characterized by increased Th cells, eosinophils, allergen-specific IgE, mast cell activation, and skin infiltration of Th2 cytokine secretion. Th2 cells produce IL-4 and IL-5, promoting antibody formation by B cells, eosinophils, and mast cells. IL-4 and IL-13 play roles in B-cell differentiation and grade conversion, thus increasing the serum IgE level in AD and triggering eosinophil and mast cell penetration of the skin [4]. Chronic AD-like skin symptoms involve predominantly Th1 cells, and chronic AD-like skin lesions exhibit inflammation-induced tissue remodeling. Th1 cells release TNF-α and IFN-γ and trigger a chronic inflammatory response [37]. In addition, epidermal barrier dysfunction results in dry and itchy skin lesions, which are aggravated by mechanical injury due to scratching. Together, these behaviors allow antigen penetration of the skin and secretion of inflammatory cytokines [38]. In this study, DNCB increased the mRNA levels of Th1 and Th2 cytokines, but this effect was reversed by IPA treatment in AD-like ear lesions.

A lot of studies have shown that TSLP acts as a master switch triggering both the onset and maintenance of AD-like skin lesions [39,40,41]. TSLP acts directly on Th2 cells to promote the differentiation of Th2 cells and is activated in the epidermis after skin-scratching action, and the increased secretion of Th2 cytokines in AD-like skin lesions results in elevated serum IgE levels. TARC, MDC, IgE and TSLP are important markers of the severity of AD-like skin symptoms. In this study, the levels of TARC, MDC, TSLP and IgE were increased in DNCB-induced AD-like skin lesions, but these effects were reversed by IPA treatment.

Because Th1 and Th2 cell differentiation and function and keratinocyte activation are important for the progression of AD-like skin lesions, an appropriate therapeutic approach to AD-like skin inflammation may be to regulate T cell and keratinocyte activation [42,43]. Keratinocytes play an important role in AD-like skin inflammation development, and TARC promotes the transport of lymphocytes to the skin. Thus, suppression of the secretion of these chemokines from keratinocytes may have therapeutic potential for AD-like skin inflammation. In this study, IPA treatment suppressed TNF-α/IFN-γ-induced increased mRNA levels of TARC, MDC and TSLP in a concentration–dependent manner.

NF-κB is an important transcriptional regulator that modulates the immune and inflammatory responses involved in the transcription of several targets, including the cytokines, chemokines and growth factors involved in the initiation of immune and inflammatory responses [44]. In addition, NF-κB is critical in the inflammatory response and mediates the activity of TARC in AD-related skin inflammation. Previous studies have reported that IPA inhibits NF-κB in various models of inflammation [14,15,16]. Our data indicate that TNF-α/IFN-γ-induced NF-κB activation was inhibited by IPA in HaCaT cells. In addition, topical application of IPA attenuated DNCB-induced NF-κB activation in mice. These results imply that IPA inhibits TARC expression by suppressing DNCB- and TNF-α/IFN-γ-induced NF-κB activation. Downregulation of these target genes is dependent on the activation of NF-κB transcription, which may explain the decreased mRNA levels of cytokines and chemokines in DNCB-induced mice and TNF-α/IFN-γ-induced cells.

MAPK signaling pathways are related to intracellular inflammatory responses. The inflammatory response promotes phosphorylation of MAPK proteins, including ERK1/2, JNK1/2, and p38 MAPK, and increases the levels of pro-inflammatory cytokines and the activation of intracellular pathways [45]. Additionally, MAPKs are implicated in the regulation of NF-κB transcriptional activation [46]. In this study, TNF-α/IFN-γ-induced phosphorylation of ERK1/2, JNK, and p38 was increased, but that of ERK1/2 was reduced by IPA treatment. Therefore, IPA treatment blocked TNF-α/IFN-γ-induced NF-κB activation by inhibiting ERK1/2 phosphorylation.

The STAT1 signaling pathway is one of the main inflammatory signaling pathways activated by several inflammatory cytokines, such as interleukins and interferons [47,48]. Upon receptor stimulation by inflammatory cytokines, a typical cell surface receptor, receptor-associated JAK2, is phosphorylated. Subsequently, STAT1 is phosphorylated and transferred into the nucleus, activating several inflammations, whereby it activates the expression of target genes. Therefore, suppression of STAT1 phosphorylation could ameliorate AD-like skin inflammatory diseases. In this study, IPA treatment suppressed the activation of STAT1 in keratinocytes, suggesting that IPA could reduce TNF-α/IFN-γ-induced STAT1 activation.

## 4. Materials and Methods

### 4.1. Reagents

IPA was provided by Professor Young Ho Kim (Chungnam National University, Daejeon, Korea). The structure of IPA is shown in Figure 1A. DNCB, hydrocortisone (HC), TNF-α, IFN-γ, dimethyl sulfoxide (*DMSO*), and MTT were from Sigma Chemical Co. (St. Louis, MO, USA). LDH was obtained from Roche (Mannheim, Germany), and BSA from RMBio (Missoula, MT, USA). Difco™ Skim Milk was purchased from BD Biosciences (Franklin Lakes, NJ, USA). Lipofectamine^®^ 2000 transfection reagent was from Invitrogen (Carlsbad, CA, USA). Nuclear factor-kappaB (NF-κB) luciferase reporter vector was obtained from Stratagene (Grand Island, NY, USA). The mouse IgE ELISA kit was from BD Biosciences (San Diego, CA, USA) and the mouse TSLP ELISA kit was purchased from R&D Systems (Minneapolis, MN, USA). PCR primers were custom synthesized by Bioneer Co. (Daejeon, Korea). Antibodies against phospho-NF-κB p65, phospho-IκBα, phospho-STAT1, STAT1, phospho-extracellular signal-regulated kinase (ERK)1/2, ERK1/2, phospho-c-Jun N-terminal kinase (JNK)1/2, JNK1/2, phospho-p38 and p38 were obtained from Cell Signaling Technology (Danvers, MA, USA). Antibodies against NF-κB p65, IκBα and β-actin were purchased from Santa Cruz Biotechnology (Santa Cruz, CA, USA). High-glucose Dulbecco’s modified Eagle’s medium (DMEM), fetal bovine serum (FBS), and penicillin–streptomycin solution were from Welgene Inc. (Gyeongsan, Korea). All chemicals and reagents were of the highest commercially available grade.

### 4.2. Animals and Treatment

Specific-pathogen-free male 5-week-old BALB/C mice were bought from Raon Bio (Yongin, Korea). The mice were allowed free access to Purina rodent chow (Purina, Seoul, Korea) and tap water and were maintained in a controlled environment at 22 ± 2 °C and 50 ± 5% relative humidity under a 12 h dark/light cycle and acclimatized for at least 2 weeks before use. Forty mice were randomly divided into five groups for the dose–response model (n = 6 mice/group): Control group, DNCB group, DNCB + 0.1% (*w/v*) IPA group, DNCB + 0.5% (*w/v*) IPA group and DNCB + 1% (*w/v*) HC group. To induce AD-like immunologic and skin lesions, DNCB was applied to the dorsal skin and ears. After complete removal of dorsal hairs within an area of approximately 8 cm^2^, 200 μL of 1% (*w/v*) DNCB solution (dissolved in a 3:1 mixture of acetone and olive oil, *v/v*) was applied for three consecutive days for sensitization. Four days after sensitization, the dorsal skin and ears were challenged with 200 μL of 0.5% DNCB solution three times per week for 7 weeks. After inducing AD, an emulsion (a 4:3:3 mixture of PEG 400, ethanol, and water, *v/v/v*) containing IPA was topically applied to the dorsal skin and ears of the mice six times per week for 4 weeks. In IPA-treated mice, IPA was topically applied 1 h before DNCB application each time. Control and DNCB-treated mice underwent topical application of an emulsion without IPA on their dorsal skin and ears. The mice were euthanized 57 days after the first application of DNCB (Figure 1B). Blood was collected from the vena cava, and the right ear was removed and subjected to histopathological analysis. The experimental protocols were approved and performed according to the regulations of the Animal Ethics Committee of Chungnam National University (CNU-01148).

### 4.3. Histopathological Analysis of Ear Tissue

The right ear tissue was removed, fixed in 10% buffered neutral formalin, embedded in paraffin, sectioned, deparaffinized, and rehydrated. Ear tissue fixed in 10% formalin was subjected to (H&E) or toluidine blue (TB) staining; various inflammatory cells and mast cells were detected to observe histopathological changes (KP&T, Cheongju, Korea). An arbitrary score was assigned to each microscopic field viewed at a magnification of 100×.

### 4.4. Measurement of Ear Thickness

Ear thickness was determined using a micrometer (Mitutoyo, Kawasaki, Japan) on the day before euthanasia [17].

### 4.5. ELISA

The serum concentrations of IgE and TSLP were assessed by sandwich ELISA using an OptEIA Mouse IgE Kit (BD Biosciences, San Diego, CA, USA) and a DuoSet Mouse TSLP Kit (R&D Systems, Minneapolis, MN, USA) according to the manufacturers’ protocols.

### 4.6. Cell Culture and Treatment

Spontaneously immortalized human keratinocytes (HaCaT; provided by Fusenig, German Cancer Research, Germany) were cultured in DMEM supplemented with 10% FBS and 1% penicillin–streptomycin at 37 °C in a humidified atmosphere containing 5% CO_2_. The cells were plated on 60-mm plates and incubated until 80% confluence. The cells were pretreated with IPA for 1 h and then with TNF-α and IFN-γ (10 ng/mL each) for 6 h. IPA was dissolved in DMSO (final concentration 0.1%) and stored at −20 °C until use.

### 4.7. Assays of Cell Viability and Cytotoxicity

Cell viability and cytotoxicity were assessed by performing MTT reduction and LDH release assays [17].

### 4.8. RNA Extraction and Quantitative Real-Time Reverse Transcription PCR

RNA extraction and cDNA synthesis were performed according to the manufacturer’s protocol. PCR was performed using primers for mouse TARC, TSLP, TNF-α, IFN-γ, IL-4, IL-5, IL-13, and GAPDH or human TARC, MDC, TSLP, and GAPDH. The primer sequences were used as previously described [17,49].

### 4.9. Transient Transfection and Luciferase Assay

Cells were transfected with the NF-κB reporter vector and Renilla luciferase reporter vector using the serum- and antibiotic-free Lipofectamine 2000 reagent. At 5 h after transfection, the transfection medium was exchanged for the complement medium. The cells were treated with IPA (1, 5, or 10 μM) for 1 h, and then stimulated with TNF-α and IFN-γ (10 ng/mL each) for 24 h. Next, the cells were lysed, and luciferase activity was determined using a luminometer (Luminoscan Ascent, Thermo Electron). The luciferase signal was normalized to that of the Renilla luciferase and expressed relative to the control value.

### 4.10. Western Blotting

HaCaT cells and mouse left-ear tissue were washed with ice-cold PBS, and proteins were isolated in 100 μL of Ceti Lysis buffer (TransLab, Daejeon, Korea) containing protease and phosphatase inhibitors. To evaluate the target protein for NF-κB p65, IκBα, ERK1/2, and STAT1 activation, they were resolved in 10% and 12% polyacrylamide gels, transferred to nitrocellulose membranes (Amersham Pharmacia Biotech, Piscataway, NJ, USA), blocked in 5% skim milk, and probed with the appropriate primary and secondary antibodies. Membranes were visualized using an enhanced chemiluminescence Western blotting detection kit (BioFact™ Co., Ltd., Daejeon, Korea).

### 4.11. Statistical Analysis

Results are expressed as means ± standard deviation (SD). Statistical significance was assessed by analysis of variance (ANOVA) followed by the Tukey–Kramer test. In vivo data are means ± SD (*n* = 6). Statistical significance was defined as ^#^ *p* < 0.05 compared to the control group or as * *p* < 0.05 compared to the DNCB-treated group. In vitro data are means ± SD (*n* = 3). Statistical significance was defined as ^#^ *p* < 0.01 compared to the control group or as * *p* < 0.01 compared to the TNF-α/IFN-γ treatment group.

## 5. Conclusions

We evaluated the inhibitory effect of IPA on AD-like skin lesions in BALB/C mice. IPA treatment suppressed DNCB-induced AD-like skin symptoms including infiltration of inflammatory and mast cells, elevated serum levels of IgE and TSLP and increased mRNA levels of TARC, MDC, TSLP, TNF-α, IFN-γ, IL-4, IL-5 and IL-13 in mice. In addition, IPA attenuated the phosphorylation of NF-κB in ear lesions and inhibited the TNF-α/IFN-γ-induced increased TARC, MDC, and TSLP level by attenuating the ERK1/2-mediated NF-κB and STAT1 activation in keratinocytes. Therefore, IPA attenuated the development of AD-like skin signs by suppressing the synthesis of cytokines and chemokines and may have therapeutic potential for AD-like skin symptoms.

## Data Availability

The data presented in this study are available on request from the corresponding author.

## Abbreviations

AD	Atopic dermatitis
DNCB	2,4-dinitrochlorobenzene
ERK1/2	Extracellular signal-regulated protein kinase
IFN-γ	Interferon-gamma
IgE	Immunoglobulin E
IL-4	Interleukin-4
IL-5	Interleukin-5
IL-13	Interleukin-13
IPA	Impressic acid
IκBα	Inhibitor of nuclear factor kappa B
MDC	Macrophage derived chemokine
NF-κB	Nuclear factor-kappa B
STAT1	Signal transducer and activator of transcription 1
TARC	Thymus and activation regulated Chemokine
TNF-α	Tumor necrosis factor-alpha
TSLP	Thymic stromal lymphopoietin
